# A Cause of Dental Caries

**Published:** 1891-01

**Authors:** 


A CAUSE OF DENTAL CARIES.—A Chicago dentist attributes the wide-spread decay of teeth in America to too much kissing. Such an outrageous theory is sufficient to produce hysterics in prim and proper spinsters, who would be unspeakably horrified if any one supposed their dental defects were due to such a cause.

